# Complement C4-A and Plasminogen as Potential Biomarkers for Prediction of Papillary Thyroid Carcinoma

**DOI:** 10.3389/fendo.2021.737638

**Published:** 2021-11-05

**Authors:** Yichao Wang, Shengliang Zhou, Dun Wang, Tao Wei, Jingqiang Zhu, Zhihui Li

**Affiliations:** ^1^ Department of Thyroid & Parathyroid Surgery Center, West China Hospital, Sichuan University, Chengdu, China; ^2^ Laboratory of Thyroid and Parathyroid Disease, Frontiers Science Center for Disease-related Molecular Network, West China Hospital, Sichuan University, Chengdu, China; ^3^ West China School of Medicine, West China Hospital, Sichuan University, Chengdu, China

**Keywords:** papillary thyroid carcinoma, biomarker, complement C4-A, plasminogen, prediction

## Abstract

**Background:**

Early diagnosis and therapy of papillary thyroid carcinoma (PTC) is essential for reducing recurrence and improving the long-term survival. In this study, we aimed to investigate the proteome profile of plasma and screen unique proteins which could be used as a biomarker for predicting PTC.

**Methods:**

Serum samples were collected from 29 PTC patients and 29 nodular goiter (NG) patients. Five PTC serum samples and five NG serum samples were selected for proteome profiles by proteomics. Eight proteins in PTC and NG serum samples were selected for confirmation by enzyme-linked immunosorbent assay analysis. Receiver operating characteristic curves was used to evaluate the diagnostic value of potential biomarkers.

**Results:**

Complement C4-A (C4A) and plasminogen (PLG) were significantly lower in serum samples of PTC patients compared with NG patients. C4A was observed to have excellent diagnostic accuracy for PTC, with a sensitivity of 91.67% and specificity of 83.33%. The diagnostic value of PLG for PTC was demonstrated by a sensitivity at 87.50% and specificity at 75.00%. The AUC for C4A and PLG was 0.97 ± 0.02 and 0.89 ± 0.05.

**Conclusion:**

C4A and PLG appeared to be excellent potential biomarkers for the prediction of PTC.

## Introduction

Thyroid carcinoma is the most common endocrine malignancy with increasing incidences worldwide (1). Epidemiologic studies have shown that thyroid cancer is responsible for 567,233 new cases and 41,071 deaths in 2018 worldwide (2). The disease may rank as the third leading cancer in women (3). Papillary thyroid carcinoma (PTC) comprises more than 80% of thyroid cancer. Cervical lymph node metastases and aggressive subset are related to the risk of recurrence or death (4, 5). The etiology of PTC is not well clarified, and its occurrence is unpredictable. Early and accurate diagnosis of PTC is essential for prevention of progression and recurrence.

Thyroid sonography and fine-needle aspiration biopsy (FNAB) are used for differentiating benign and malignant thyroid lesion. However, FNAB is invasive and may yield 20% indeterminate cytology (6). Many molecular testing techniques have been made to aid in the diagnosis of PTC, such as ThyroSeq mutational panel, the Afirma gene expression classifier, immunohistochemical stains, and proteomic analysis (7, 8). However, these tests are based on biopsy samples and incapable of timely monitoring the occurrence of PTC. Therefore, accurate and timely biomarkers of serum protein are required to predicting PTC. With respect to serum protein for distinguishing PTC, Hu et al. (9) showed that serum vascular adhesion protein-1 with a 66.7% sensitivity and 77.4% specificity was profoundly downregulated in thyroid cancer group which included PTC and follicular thyroid carcinoma. Lu et al. (10) reported that serum complement C4-A/B increased in PTC patients by using weak cation exchange magnetic beads fractionation followed by matrix-assisted laser desorption/ionization–time-of-flight mass spectrometry. However, the specificity and sensitivity for diagnosis is not high enough.

We aimed to screen for novel serum proteomic biomarkers for predicting PTC. Through screening assay using proteomics and enzyme-linked immunosorbent assay analysis (ELISA), we successfully identified that serum C4A and plasminogen (PLG) were proteins that met these requirements.

## Materials and Methods

### Study Population

A total of 29 PTC patients and 29 nodular goiter (NG) patients were enrolled in this study between September 2018 and July 2019. Twenty-nine PTC patients included 27 patients diagnosed as primary thyroid malignancy and two as suspicious for malignancy according to cytology result of FNAB. Twenty-nine NG patients with a maximum diameter of larger than 4 cm estimated by ultrasonography underwent thyroidectomy based on current Chinese guidelines. Pathological diagnosis with PTC or NG was confirmed by the histopathological result of resected specimens. We excluded those patients combined with other cancer types. The clinical characteristics and demographic information were collected from medical records, such as age, gender, tumor node metastasis (TNM) status, and multiplicity of tumor. The baseline characteristics of PTC cases and NG cases are presented in **Tables 1**, **2**. We excluded patients with comorbid conditions such as other forms of cancer and inflammatory diseases. The present study was approved by the ethics committee of the West China Hospital of Sichuan University, and all patients enrolled in the study signed written informed consent.

**Table 1:** Detailed information of characteristics of study samples used in proteomics.

Number	Age (year)	Sex	TNM classification	Multiplicity	Diagnosis
1	49	Female	T2N0M0	No	PTC
2	59	Female	T1aN0M0	No	PTC
3	45	Male	T1bN1aM0	No	PTC
4	33	Female	T3bN1aM0	No	PTC
5	49	Female	T1aN0M0	No	PTC
6	40	Female	–	Yes	NG
7	40	Female	–	Yes	NG
8	38	Male	–	Yes	NG
9	47	Female	–	No	NG
10	34	Female	–	Yes	NG

PTC, papillary thyroid carcinoma; NG, nodular goiter.

**Table 2:** Detailed information of characteristics of study samples used in ELISA.

Number	Age (year)	Sex	TNM classification	Multiplicity	Diagnosis
1	40	Female	T1bN1aM0	No	PTC
2	53	Male	T1aN1aM0	No	PTC
3	43	Female	T3bN1bM0	No	PTC
4	28	Female	T1aN1bM0	No	PTC
5	22	Female	T3aN1bM0	Yes	PTC
6	39	Female	T2N1aM0	Yes	PTC
7	52	Female	T3bN1aM0	No	PTC
8	28	Female	T1aN1aM0	Yes	PTC
9	28	Female	T3aN1aM0	Yes	PTC
10	26	Female	T3bN1aM0	Yes	PTC
11	26	Female	T2N1aM0	No	PTC
12	38	Male	T3bN1aM0	No	PTC
13	54	Male	T1bN1aM0	Yes	PTC
14	27	Female	T1aN0M0	No	PTC
15	53	Female	T1aN0M0	No	PTC
16	30	Female	T1bN1aM0	No	PTC
17	53	Female	T1aN0M0	No	PTC
18	52	Male	T3bN1aM0	No	PTC
19	43	Female	T3bN1aM0	No	PTC
20	30	Female	T1bN1aM0	No	PTC
21	34	Female	T2N0M0	No	PTC
22	61	Female	T1aN0M0	No	PTC
23	31	Female	T3N1bM0	No	PTC
24	33	Female	T1aN1aM0	No	PTC
25	49	Female	–	Yes	NG
26	58	Male	–	Yes	NG
27	58	Female	–	Yes	NG
28	36	Female	–	Yes	NG
29	49	Female	–	No	NG
30	46	Female	–	No	NG
31	61	Female	–	Yes	NG
32	36	Female	–	No	NG
33	47	Female	–	Yes	NG
34	48	Female	–	Yes	NG
35	52	Female	–	No	NG
36	61	Female	–	Yes	NG
37	72	Female	–	Yes	NG
38	61	Female	–	Yes	NG
39	57	Female	–	No	NG
40	85	Female	–	Yes	NG
41	35	Female	–	Yes	NG
42	63	Female	–	Yes	NG
43	45	Female	–	No	NG
44	57	Female	–	No	NG
45	38	Male	–	Yes	NG
46	66	Female	–	No	NG
47	62	Female	–	Yes	NG
48	22	Female	–	Yes	NG

ELISA, enzyme-linked immunosorbent assay; PTC, papillary thyroid carcinoma; NG, nodular goiter.

### Sample Collection

Five-milliliter peripheral venous blood sample was collected prior to any therapeutic intervention by EDTA-containing vacutainer tube. Samples were subjected to centrifugation at 1,600 rpm and 4°C for 10 min. The supernatant was aliquoted and stored at −80°C until use.

### Screening Assay Using Proteomics

Five PTC serum samples and five NG serum samples were used to perform the screening assay by using proteomics. The proteomics were performed by Shanghai OE Biotech. Co., Ltd. according to the manufacturer’s suggested instruction. Briefly, 10 µl serum sample was added to the resin slurry in the column, then incubated with gentle shaking for 60 min. Column was added to a 2-ml collection tube and centrifuge at 1,000×*g* for 2 min. The filtrate was collected for further processing. Protein concentration was determined using a bicinchonininc acid protein assay (11). The protein was digested into peptides using filter-aided sample preparation. The digested peptides were desalted using C18-Reverse-Phase SPE Column. Liquid chromatography (LC)-data-dependent acquisition (DDA)/data-independent acquisition (DIA)-mass spectrometry (MS)/MS was performed by a QE mass spectrometer (Thermo, Waltham, MA, USA) equipped with an Easyspray source (Thermo, USA). The LC-MS/MS raw data were imported in Maxquant for labeling free quantification analysis. RAW files were imported into Spectronaut pulsar X to generate the spectral library. The DIA data were analyzed with Spectronaut Pulsar X.

### Enzyme-Linked Immunosorbent Assay

The levels of synaptic vesicle membrane protein VAT-1 homolog (fragment) (VAT1), protein Z-dependent protease inhibitor (SERPINA10), apolipoprotein B-100 (APOB), complement factor I (CFI), complement component C1q receptor (CD93), C4A, extracellular matrix protein 1 (ECM1), and PLG in serum were detected using commercial kits purchased from RayBiotec, Inc (Norcross, GA, USA), Novus Biologicals (Centennial, CO, USA), and Abbexa Ltd (Cambridge, UK). The assay procedure was conducted by following the manufacturer’s protocols. Diluted serum samples were each added into 96-well plate precoated with an antibody. Plates were incubated at 37°C or room temperature with gentle shaking. After washing, tetramethylbenzidine (TMB) substrate solution were added into each well followed by incubation for 10–30 min at room temperature or 37°C. After terminating the reactions, the absorbance of each well was recorded on a spectrophotometer (Tecan Trading, Mannedorf, Switzerland) at 450 nm.

### Statistical Analysis

Continuous variables with a normal distribution were expressed as the mean ± standard deviation (SD). All statistical analyses were assessed by the Student’s *t*-test using GraphPad Software Prism 5 (San Diego, CA, USA). A value of *p* < 0.05 was considered statistically significant. The sensitivity and specificity of C4A and PLG for diagnosis of PTC was evaluated by receiver operating characteristic (ROC) curve and the area under the curve (AUC). The ROC curves with an AUC ≥0.8 demonstrate good discriminatory power.

## Results

### Screening of Serum Samples

The serum protein spectrum in five PTC and five NG patients were screened using proteomics. The analysis showed that four proteins were upregulated while eight proteins were downregulated in PTC group compared with NG group (**Table 3**). Among them, we selected eight proteins for further confirmation analysis, including four upregulated and four downregulated with *p*-value ≤0.01.

**Table 3:** Identification information for differentially expressed proteins between PTC and NG.

Protein name	Gene name	Fold change	*p*-Value	Regulation
Synaptic vesicle membrane protein VAT-1 homolog (fragment) (VAT1)	*VAT1*	3.21	0.03	Up
Protein Z-dependent protease inhibitor (SERPINA10)	*SERPINA10*	1.67	0.01	Up
Apolipoprotein B-100 (APOB)	*APOB*	1.56	0.01	Up
Complement factor I (CFI)	*CFI*	1.54	0.02	Up
Complement component C1q receptor (CD93)	*CD93*	0.52	0.01	Down
Complement C4-A (C4A)	*C4a*	0.55	0.01	Down
Extracellular matrix protein 1(ECM1)	*ECM1*	0.62	0.01	Down
Plasminogen (PLG)	*PLG*	0.63	0.01	Down
Coagulation factor XIII A chain	*F13A1*	0.45	0.02	Down
Platelet factor 4	*PF4*	0.53	0.02	Down
Insulin-like growth factor-binding protein complex acid labile subunit	*IGFALS*	0.62	0.03	Down
Apolipoprotein F	*APOF*	0.28	0.04	Down

PTC, papillary thyroid carcinoma; NG, nodular goiter.

### Selected Protein Levels in PTC and NG Plasma

**Figure 1:**
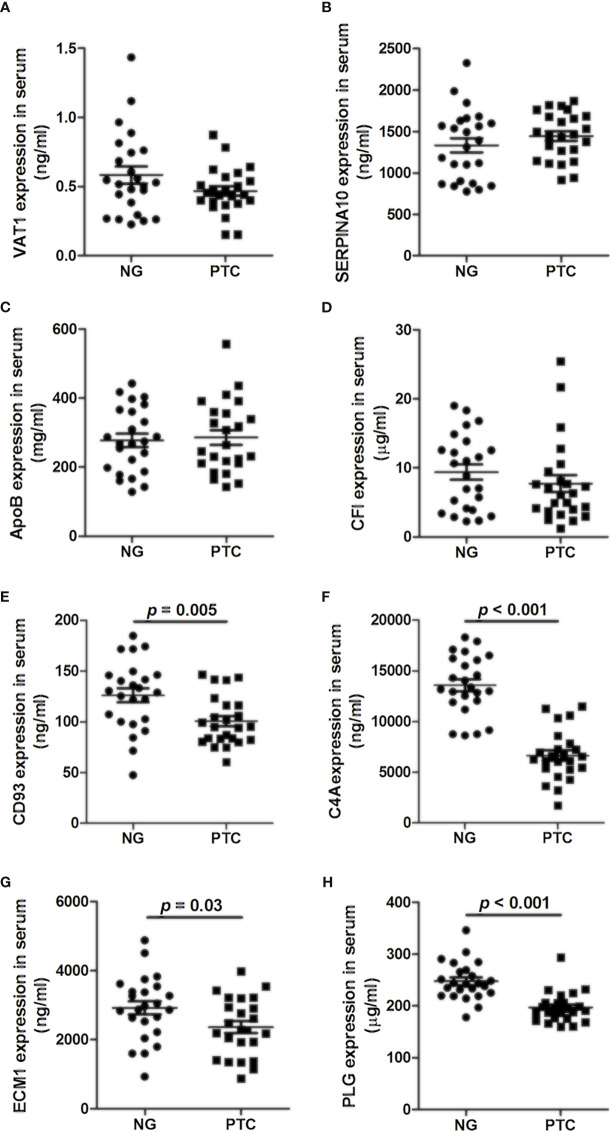
Concentrations of **(A)** VAT1, **(B)** SERPINA10, **(C)** APOB, **(D)** CFI, **(E)** CD93, **(F)** C4A, **(G)** ECM1, and **(H)** PLG in the plasma of PTC and NG.

The levels of VAT1, SERPINA10, APOB, CFI, CD93, C4A, ECM1, and PLG were analyzed by ELISA in 24 PTC samples and 24 NG samples. We found that VAT1, SERPINA10, APOB, and CFI exhibited no significant differences between the PTC and NG samples (**Figures 1A**–**D**). As shown in **Figures 1E**–**H**, the expression levels of CD93, C4A, ECM1, and PLG in the PTC group were significantly decreased compared with the NG group (*p* < 0.05).

### Diagnostic Value of Serum C4A and PLG for PTC

**Figure 2:**
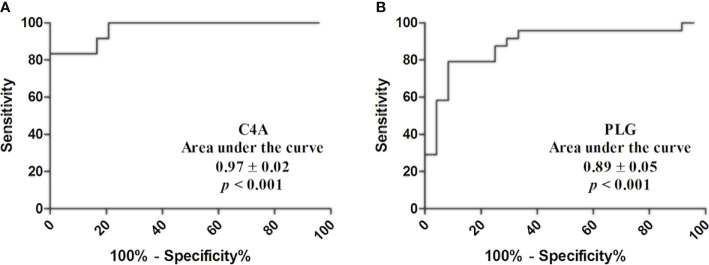
Diagnostic value of C4A and PLG for PTC. **(A)** Receiver operating characteristic curves of C4A. **(B)** Receiver operating characteristic curves of PLG.

Based on the quantitative results, the diagnostic values of serum C4A and PLG for PTC was evaluated by ROC curve. The cutoff values were chosen by considering the maximum sensitivity and specificity for the diagnosis of PTC. As shown in **Figure 2** and **Table 4**, the AUC for C4A was 0.97 ± 0.02. When the cutoff was set at 10,884 ng/ml, the sensitivity of C4A was 91.67% and the specificity reached 83.33% (**Figure 2A**). In addition, the AUC for PLG was 0.89 ± 0.05. PLG was also observed to have an excellent diagnostic accuracy for PTC with a respective sensitivity and specificity of 87.50% and 75% for a cutoff value of 225.2 μg/ml (**Figure 2B**).

**Table 4:** Diagnostic value of C4A and PLG for PTC.

	Cutoff	Sensitivity (95% CI)	Specificity (95% CI)	Likelihood ratio
C4A	10,884 ng/ml	91.67 (73.00–98.97)	83.33 (62.62–95.26)	5.50
PLG	225.2 μg/ml	87.50 (67.64–97.34)	75.00 (53.29–90.23)	3.50

C4A, complement C4-A; PLG, plasminogen; PTC, papillary thyroid carcinoma; CI, confidence interval.

## Discussion

To the best of our knowledge, this is the first study investigating the expression levels of serum C4A and PLG in PTC and NG plasma by proteomics and ELISA. Based on our study, we found C4A and PLG levels were significantly lower in the PTC group compared with the NG group. C4A and PLG were observed to have an excellent diagnostic accuracy for PTC with high sensitivity and specificity. These findings implied that serum C4A and PLG may be potential biomarkers for predicting PTC. It is known that FNAB is frequently used in clinics for evaluating thyroid nodules. Hahn et al. reported FNAB for the prediction thyroid malignancy at presentation with a sensitivity of 89.6% and specificity of 98.7%, respectively (12). Nonetheless, this method is invasive. In addition, molecular testing of FNA samples, such as RNA test and DNA-RNA test, is recommended for indeterminate cytology (13), suggesting the molecular testing technique is not a routine recommendation for evaluating thyroid nodule. Livhits et al. reported that RNA test (Afirma genomic sequencing classifier) showed a sensitivity of 100% and a specificity of 79.6% for diagnosing indeterminate thyroid nodules (ITN). The sensitivity of DNA-RNA test (ThyroSeq v3 multigene genomic classifier) for diagnosing ITN reached 96.9% with a specificity of 84.8% (14). However, the subjects included were thyroid FNAB specimen. This biopsy is an invasive procedure. In the present study, peripheral venous blood sample were examined *via* proteomics and ELISA analysis. C4A was observed to have excellent diagnostic accuracy for PTC, with a respective sensitivity and specificity of 91.67% and 83.33%. The sensitivity and specificity of PLG was 87.50% and 75.00% for PTC diagnosis, respectively.

C4A is one of inflammatory mediators which plays a role in the formation of immune complexes and regulation of binding of immune aggregates (15, 16). In addition, C4 was observed high expression in the thyroid gland compared with the heart, pancreas, and thymus (17). Several studies have surveyed that abnormal C4A expression is associated with some cancers. Tikhonov et al. reported that serum C4A was associated with colorectal cancer developing (16). Zhang et al. reported that the levels of serum C4A in chemosensitive for epithelial ovarian cancer group were significantly higher than in the chemoresistant group (18). Broek et al. reported that the upregulation of serum C4A was associated with breast cancer (19). Lu et al. identified the upregulation of serum C4A/B in PTC compared with benign thyroid node and healthy individuals (10). In contrast, we found that serum C4A in PTC group was significantly decreased with high sensitivity of 91.67% and specificity of 83.33% compared with the NG group. This result might be attributed to the different comparison. However, the mechanism of C4A affecting the tumorigenesis of PTC has not been demonstrated. Further mechanistic analysis is required to understand the biological role of C4A.

PLG, a 92–94-kilodalton protein produced in the liver and circulated in the blood, is known to be involved in the degradation of extracellular matrix, cell migration, angiogenesis, oncogenesis, and metastasis (20, 21). The PLG is associated autophagy of cancer (22). Fang et al. demonstrated that Plasminogen kringle 5 suppressed the growth gastric cancer by inhibiting angiogenesis and apoptosis (23). Yang et al. found that plasminogen kringle 5 can inhibit hepatocellular carcinoma *via* antiangiogenic activity (24). Tykhomyrov et al. reported that PLG-treated lung adenocarcinoma cells exhibited autophagy induction by upregulation of beclin-1 levels (25). In the present study, we observed that PTC group showed a great downregulation of PLG compared with the NG group, providing insight into the association between PLG and PTC. However, the mechanism by which PLG affects PTC is unknown. Further studies are warranted to exemplify.

Of course, we have to admit several limitations in present study. First, a small number of PTC and NG samples were included in the study. Second, due to financial constraints, we did not investigate serum protein in healthy controls. Third, we have not analyzed how C4A and PLG are involved in tumorigenesis of PTC. Therefore, well-designed trials with larger samples are required to confirm that C4A and PLG may be potential biomarkers for the diagnosis of PTC.

In conclusion, this study reveals that serum C4A and PLG may be potentially useful biomarkers for the prediction of PTC. C4A and PLG with a high sensitivity and specificity appear to be developed into a bedside test for rapidly predicting PTC.

## Data Availability Statement

The datasets presented in this study can be found in online repositories. The names of the repository/repositories and accession number(s) can be found below: Proteomexchange [accession: number:PXD029304].

## Ethics Statement

The studies involving human participants were reviewed and approved by the ethics committee of the West China Hospital of Sichuan University. The patients/participants provided their written informed consent to participate in this study.

## Author Contributions

ZL, JZ, and TW designed the research. YW, SZ, and DW performed the experiments and carried out statistical analysis. YW drafted the article. All authors contributed to the article and approved the submitted version.

## Funding

This work was supported by Sichuan Science and Technology Program (grant number 2019YJ0038).

## Conflict of Interest

The authors declare that the research was conducted in the absence of any commercial or financial relationships that could be construed as a potential conflict of interest.

## Publisher’s Note

All claims expressed in this article are solely those of the authors and do not necessarily represent those of their affiliated organizations, or those of the publisher, the editors and the reviewers. Any product that may be evaluated in this article, or claim that may be made by its manufacturer, is not guaranteed or endorsed by the publisher.

## Abbreviations

PTC, papillary thyroid carcinoma; NG, nodular goiter; ELISA, enzyme-linked immunosorbent assay analysis; C4A, complement C4-A; PLG, plasminogen; TNM, tumor node metastasis; VAT1, synaptic vesicle membrane protein VAT-1 homolog (fragment); SERPINA10, protein Z-dependent protease inhibitor; APOB, apolipoprotein B-100; CFI, complement factor I; CD93, complement component C1q receptor; ECM1, extracellular matrix protein 1; ITN, indeterminate thyroid nodules.
